# Heat and dehydration induced oxidative damage and antioxidant defenses following incubator heat stress and a simulated heat wave in wild caught four-striped field mice *Rhabdomys dilectus*

**DOI:** 10.1371/journal.pone.0242279

**Published:** 2020-11-13

**Authors:** Paul J. Jacobs, M. K. Oosthuizen, C. Mitchell, Jonathan D. Blount, Nigel C. Bennett

**Affiliations:** 1 Department of Zoology and Entomology, Mammal Research Institute, University of Pretoria, Pretoria, South Africa; 2 Centre for Ecology and Conservation, College of Life & Environmental Sciences, University of Exeter, Penryn, Cornwall, United Kingdom; Universidade de Brasilia, BRAZIL

## Abstract

Heat waves are known for their disastrous mass die-off effects due to dehydration and cell damage, but little is known about the non-lethal consequences of surviving severe heat exposure. Severe heat exposure can cause oxidative stress which can have negative consequences on animal cognition, reproduction and life expectancy. We investigated the current oxidative stress experienced by a mesic mouse species, the four striped field mouse, *Rhabdomys dilectus* through a heat wave simulation with *ad lib* water and a more severe temperature exposure with minimal water. Wild four striped field mice were caught between 2017 and 2019. We predicted that wild four striped field mice in the heat wave simulation would show less susceptibility to oxidative stress as compared to a more severe heat stress which is likely to occur in the future. Oxidative stress was determined in the liver, kidney and brain using malondialdehyde (MDA) and protein carbonyl (PC) as markers for oxidative damage, and superoxide dismutase (SOD) and total antioxidant capacity (TAC) as markers of antioxidant defense. Incubator heat stress was brought about by increasing the body temperatures of animals to 39–40.8°C for 6 hours. A heat wave (one hot day, followed by a 3-day heatwave) was simulated by using temperature cycle that wild four striped field mice would experience in their local habitat (determined through weather station data using temperature and humidity), with maximal ambient temperature of 39°C. The liver and kidney demonstrated no changes in the simulated heat wave, but the liver had significantly higher SOD activity and the kidney had significantly higher lipid peroxidation in the incubator experiment. Dehydration significantly contributed to the increase of these markers, as is evident from the decrease in body mass after the experiment. The brain only showed significantly higher lipid peroxidation following the simulated heat wave with no significant changes following the incubator experiment. The significant increase in lipid peroxidation was not correlated to body mass after the experiment. The magnitude and duration of heat stress, in conjunction with dehydration, played a critical role in the oxidative stress experienced by each tissue, with the results demonstrating the importance of measuring multiple tissues to determine the physiological state of an animal. Current heat waves in this species have the potential of causing oxidative stress in the brain with future heat waves to possibly stress the kidney and liver depending on the hydration state of animals.

## Introduction

Extreme temperature climatic events (heat waves) are a real threat to animal biodiversity through a variety of lethal and sublethal effects [1–4]. Lethal heat stress from heat waves are likely due to dehydration and cellular heat damage [5], with just a single day of extreme temperatures leading to a mass die-off of an endangered bird the Carnaby’s Cockatoo (*Calyptorhynchus latirostris*) [6]. Several other mass die-off events have occurred in the last 20 years resulting in the deaths of humans, bats and birds [4, 7–9]. Sublethal effects of repeated exposure to extreme heat events may include loss of body condition, compromised reproduction and reduced cognitive performance, which can result in overall population declines [3]. These heat waves are predicted to become more frequent and intense in the Anthropocene [8, 10–12], highlighting concerns for species extinctions [13].

Small animals are generally assumed to circumvent the effects of climate change due to the use of microsites within a habitat to escape extreme temperatures [14–17]. In addition to the use of microsites, smaller animals have a larger surface area to volume ratio allowing for rapid heat loss assuming air temperature is below skin temperature [18]. A larger surface area to volume ratio can also be detrimental since rapid heat loss is accompanied by rapid heat gain and without the presence of microsites may drastically compromise small animal survival [14].

Animals can behaviourally alleviate the effects of heat stress, through drinking more water [19–21], reduce thermogenic activity by eating less [22, 23] and lowering activity rate [24, 25]. Rodents do not sweat (except from their footpads) [26, 27], or pant to increase evaporative water loss [28]. Instead, rodents primarily use saliva spreading for evaporative heat loss, while rodent species that do not utilise saliva spreading suffer exaggerated responses to heat stress [20].

One pattern observed as a consequence of anthropogenic climate change, is the geographic shift of species to escape elevated temperatures [10]. One southern African species, the mesic four-striped field mouse, *Rhabdomys dilectus* (de Winton, 1987), has been proposed to be at risk and may undergo a geographic shift to escape climate change [29, 30]. The study species prefers the grassland and savannah biomes with ground cover and water [19], with the ground cover providing a thermal buffer to avoid extreme temperatures [29]. The congeneric desert living four striped field mouse *Rhabdomys pumilio* (Sparrman 1784) has a thermoneutral zone (TNZ) of 32°C [31], with the mesic species’ TNZ still to be determined.

Aerobic organisms constantly produce reactive oxygen species (ROS) from metabolism, and utilise antioxidants to reduce excessive ROS in order to maintain redox balance [32, 33]. Despite the negative connotation to ROS, ROS are important to cellular signalling [34], to inflammation response [35], altering glucose uptake and metabolism [36], immune response [37], allowing for the preparation to deal with hypoxic stress [38] and osmoprotective signalling [39].

Heat stress and dehydration independently can disrupt this balance [40–42]. Heat stress can disrupt this balance through excessive metabolic ROS production and lowered antioxidant activity, resulting in a state of oxidative stress [43, 44]. Dehydration disrupts this balance caused by hyperosmolality induced from cellular shrinkage and compromised membrane functionality [45–47]. This excessive oxidative imbalance in favour of ROS can ultimately lead to oxidative damage to DNA [48, 49], lipids [50] and proteins [51, 52]. Oxidative stress can reduce cognitive and motor performance [53], fertility [54, 55] and life expectancy [56, 57]. Therefore, biomarkers of oxidative stress can provide highly relevant insights into the physiological state of an organism [58, 59], making it possible to establish whether an animal is vulnerable to changes in its environment. However, we are not aware of any previous studies that have measured oxidative stress levels in a variety of tissues as a consequence of exposure to different thermal regimes in the four striped field mouse.

We measured the heat stress response in terms of oxidative damage and antioxidant defense of four-striped field mouse following an incubator heat stress and dehydration experiment and a simulated heat wave. The incubator heat stress is a whole-body fever range exposure, which is a representation of an animal’s upper limit and failing thermoregulatory system. The simulated heat wave represents current heat wave extremes where the animals were caught (collected and simulated weather station data). Oxidative damage and antioxidant defense were measured in three organs, namely the liver, kidney and brain. These three tissues account for over 60% of the body’s resting metabolic rate, at least in humans [60, 61]. Brain was chosen for its susceptibility to thermal stress, and in particular its involvement in multiple organ dysfunction during heat stress [62, 63]. The kidney was selected based on its importance in water retention and an abundance of long-chain polyunsaturated fatty acids in the composition of renal lipids [64], such molecules being especially susceptible to lipid peroxidation [65]. Lastly, liver was chosen because it is an important source of glutathione [66], the most important antioxidant in determining total antioxidant capacity (TAC) in tissues [67]. In addition to the liver’s relevance to TAC, heat stress is also associated with elevated oxidative damage and antioxidant defense in this tissue [68, 69], due in part to fluctuations in labile iron [70].

We predicted that the four-striped field mouse would demonstrate increased susceptibility to heat stress induced oxidative stress in the incubator experiment as compared to the simulated heat wave relative to their respective controls. In this context we define oxidative stress as significant changes in oxidative damage and antioxidant defense compared to the controls.

Oxidative damage and antioxidant defense occur concomitantly, therefor increases in antioxidant defense (superoxide dismutase (SOD) enzyme or TAC) will demonstrate increased oxidative stress, whereas oxidative damage (lipid and/or proteins) will represent a compromised antioxidant defense.

## Methods

Animal Ethics Committee University of Pretoria (AEC) with approval number EC008-17.

### Animal maintenance

Ten adult male four-striped field mice, were wild caught and used in the incubator heat stress experiment and thirteen adult male animals were wild caught for the simulated heatwave. Males were used in order to prevent sex differences in oxidative stress [71–73]. The field mice were captured at the Rietvlei nature reserve (3800ha, Centurion, South Africa, -25° 53' 29.39" S, 28° 17' 22.80" E), using metal Sherman traps (26 cm x 9 cm x 9 cm), baited with a mixture of oats and peanut butter. Rietvlei is a local nature reserve and we obtained written permission from the manager to perform work here. Mice used for the incubator heat stress experiment were caught between March 2017-June 2017; whereas those for the simulated heat wave experiment were captured between January 2019-March 2019. Once caught, mice were maintained in field cages and subsequently transported to the Zoology and Entomology Department, at the University of Pretoria. The mean body mass before the experiment of the striped field mouse used for the incubator heat stress experiment was 62.7 ± 16.4 standard deviation (SD) g, whereas the mean body mass before the experiment for the simulated heat wave experiment was 43.1 ± 5.6 (SD) g. The mice were weighed weekly to assess body condition. Mice were weighed to ± 0.1 g prior to and after an experiment. Animals were housed in a room at the University of Pretoria and acclimated to a 12L:12D photoperiod, 40% RH and temperature of around 23°C. This temperature also resembles the average temperature experienced during a summer day. Mice were maintained in captivity for at least 60 days prior to their use for both experiments. This initial acclimation period was performed to minimise the influence of stress by bringing wild animals into captivity. All mice were housed individually in 40 x 25 x 12 cm standard laboratory mouse containers lined with wood shavings and a rock, toilet rolls, a small plastic container for a nest, and with tissue paper for nesting material. Animals were provided *ad libitum* with water and food. Food was provided every second day in the form of sunflower seeds, corn, banana, carrot, apple shavings or sweet potato slices. The cages were cleaned weekly.

### Experiment 1: Incubator heat stress experimental design and protocol

The heat stress treatment was used to investigate the oxidative markers following a 6-hour whole-body fever range hyperthermia acute heat stress (39–40.8°C body temperature (T_b_)) and dehydration stress in an incubator. This incubator heat stress protocol followed that of Ostberg, Kaplan [74], a period of 6-hour of T_b_ around 39–40.8°C T_b_ was used to induce whole-body fever range hyperthermia that could result in oxidative heat stress response.

A control group maintained at an ambient temperature (T_a_) of 25°C (animal core T_b_ averaged ±36.6°C) served to determine oxidative markers without the influence of heat stress. Individuals were randomly allocated to the heat-stressed group and the control group. Lastly, food and water were not provided during this experiment to prevent metabolic and humidity increases respectively, which will influence heat loss.

In order to determine animal core T_b_, temperature-sensitive PIT tags (BioTherm, Identipet), which can be read with a PIT tag reader, were injected intraperitoneally using sterile syringes at least a week prior to the experiment. Due to the length of the experiment, all individuals were injected with 1ml of saline on day 1 of the experiment just prior to the thermal manipulation to help animals buffer against dehydration over the 6-hour time period.

The incubator was pre-heated to 41°C T_a_ (heat stress) or 25°C T_a_ (control) prior to the animal being placed inside the incubator. Animals were subsequently transferred to an experimental chamber of 25 x 13 x 16 cm, and placed in the incubator. Lights were switched off inside the incubator to minimise extra heat from light sources inside the incubator. Animal core T_b_ was monitored using a PIT reader antenna; incubator temperature (T_a_) was modulated as required to maintain T_b_ at around 39–40.8°C, the required range (S1 Table). Readers were set to record every 10 seconds. After 6-hours, animals were removed from the experiment.

### Experiment 2: Simulated heat wave experimental design and protocol

#### Control, transition and heat wave simulation

**Fig 1:**
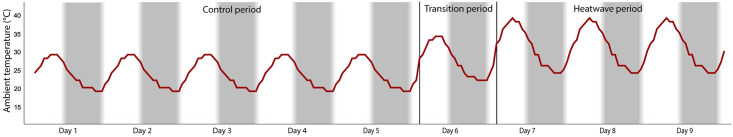
Photoperiod and temperature profile used to simulate control, transition, and heat wave temperatures. The white areas represent day time and shaded areas represent night time. The black lines represent changes in experimental condition, with the control temperatures lasted 5 days, transition temperatures lasted 1 day and the simulated heat wave lasted 3 days. The red line represents the temperature cycle that animals were exposed to during the 9-day experiment.

The four striped field mice were transferred to large plastic individual containers (60 x 40 x 30 cm) lined with wood shavings, a small plastic container for a nest and a toilet roll and tissue paper provided as nesting material. Lighting was set to a 14L:10D long day schedule, which included 4 hours of ‘twilight’ with increasing and decreasing light intensities simulating dawn and dusk respectively. The long day photoperiod was accompanied by a typical temperature cycle, which was determined through climatic data obtained from the South African weather service (Fig 1).

The control group of mice were set up to determine the influence of a daily temperature cycle from an average summer day (calculated from the South African Weather service data) to simulate minimal to no heat stress at least when compared to a heat wave. Control mice were maintained for nine days on a cycle that oscillated between a minimum of 19°C and a maximum of 29°C (Fig 1). In contrast, for the heat wave simulation, all animals were first maintained at control temperatures for five days, then transitioned to temperatures that oscillated between a minimum of 22°C and a maximum of 34°C for one day, followed by heat wave temperatures which oscillated between a minimum of 24°C and a maximum of 39°C for a period of full three days. During changes in the temperature (control to transition, and transition to heat wave), temperatures were set to represent the new treatment condition starting at 9:00 am. During experimentation, the experimenter influence was kept to a minimum, with a 30 min interval (between 8:00 am-8:30 am) for feeding and checking the general welfare of the animals. The mice were given a fixed amount of water and food (sunflower seeds every day, fresh fruit/vegetables every second day).

### Calculation of simulated heat wave temperatures

Weather station data provided by the South African Weather Service were corrected using a temperature-humidity index (THI). This index was calculated from the wet and dry bulb air temperatures for a particular day according to the following formula: THI = 0.72 (W + D) + 40.6, where W is wet bulb and D is dry bulb temperature in degree centigrade. The following website was used to convert climatic data values to heat index values (http://www.wpc.ncep.noaa.gov/html/heatindex.shtml). A THI was used to correct for constant humidity inside the temperature control rooms such that animals would feel the perceived air temperatures as actual temperature conditions, instead of elevated temperatures with higher humidities.

### Euthanasia and tissue excision

All mice were euthanised with an overdose of isoflurane immediately at the end of each respective experiment. All samples were collected at the same time to prevent daily rhythm effects, with tissues collected in the same order within 10 minutes at post-mortem with an approximate 1-minute interval between tissues. This was done to prevent and/or minimise proteins and metabolites from denaturing from dissection to being flash frozen. The liver, kidney, and brain were collected and flash-frozen in liquid nitrogen, and subsequently stored at -80°C until analysis (less than 6 months for all tissues).

### Analyses of oxidative damage and antioxidant defense

#### Tissue homogenization

Tissues (liver, brain and kidney) were homogenised on ice by 10% weight per volume in 20 mM HEPES (N-2 hydroxyethylpiperazine-N9-2-ethanesulfonic acid) buffer on an Ultra Turrax T18 Basic Homogenizer (IKA, Staufen, Germany) for the incubator heat stress and on an Ultra Turrax T25 Basic Homogenizer (IKA Labortechnik, Germany) for the simulated heat wave experiment. Homogenates were then stored in a -80°C freezer until the time of analysis (less than 6 months for all tissues).

#### Malondialdehyde: Incubator heat stress and simulated heatwave

The concentrations of MDA in all tissue homogenates (i.e. liver, kidney and brain) were measured by high-performance liquid chromatography (HPLC) using standard techniques [75]. Prepared samples were injected 20 μL into an Agilent HPLC system (InfinityLab Solutions, California, USA) fitted with a 5 μm ODS guard column and a Hewlett-Packard Hypersil 5 μ ODS 100 x 64.6 mm column maintained at 37°C. The mobile phase was methanol-buffer (40:60, v/v; 50 mM anhydrous solution of potassium monobasic phosphate at pH 6.8), running isocratically over 3.5 min at a flow rate of 1 ml per min. Data were collected using a fluorescence detector (RF2000; Dionex) set at 515 nm (excitation) and 553 nm (emission). For calibration, a standard curve was established using a TEP stock solution (5 μM in 40% ethanol) serially diluted using 40% ethanol. Results are expressed as μMol MDA per g homogenate.

#### Protein carbonyl: Incubator heat stress

PC concentrations were measured from tissue homogenates (i.e. liver, kidney, and brain). Oxidation or oxidative cleavage of proteins results in the production of carbonyl groups following standard technique [76], which covalently react with 2,4-dinitrophenylhydrazine (DNPH) to form 2,4-dinitrophenyl (DNP) hydrazone. DNP is detected via spectrophotometry at a wavelength of 370 nm [77]. Our study protocol differed by using 1ml of 20% TCA instead of 125μL of 50% TCA. Absorbances were read using a Spectramax M2 plate reader (Molecular Devices Corp., Sunnyvale, CA, USA). Samples were run in duplicate with a repeatability of *r* = 0.99 between control and samples. Protein content was determined using the Bradford assay using a bovine serum albumin (BSA) standard curve. 180 μL of guanidine-HCL solution was added to 20 μL of control sample (HCL solution) in a 1:10 ratio. Absorbances were read at 280nM using a Spectramax M2 plate reader (Molecular Devices Corp., Sunnyvale, CA, USA). Samples for the Bradford assay were run in duplicate with a repeatability of *r* = 0.99. The results are expressed in μMol per g protein.

#### Protein carbonyl: Simulated heat wave

PC was measured from the tissue homogenates (i.e. liver, kidney, and brain). PC concentrations were measured using a commercially available kit (Sigma-Aldrich, cat. no. MAK094, MO, USA), reading the absorbance of samples using an Eon high-performance microplate spectrophotometer (BioTek Instruments Inc., USA). Protein content of each sample was analysed using a BCA assay (Sigma-Aldrich, cat. no. BCA1 and B9643, MO, USA) using a BSA standard (Sigma-Aldrich, cat. no. P0914, MO, USA). PC samples were run in duplicate with a repeatability of *r* = 0.70. BCA samples were run in duplicate with a repeatability of r = 0.83. Results are expressed as μMol per g protein.

#### Superoxide dismutase: Incubator heat stress and simulated heatwave

SOD activity was measured in all tissue homogenates (i.e. liver, kidney and brain). SOD is an enzymatic antioxidant that catalyses the dismutation of superoxide anions to oxygen and hydrogen peroxide [78]. Analyses were performed following standard techniques [79]. SOD content was measured with a commercially available kit (Superoxide Dismutase Assay Kit, Cayman Chemical Co., Ann Arbor, MI, USA) that measures the percentage of superoxide radicals that undergo dismutation in a given sample. Absorbance was read at 450 nm using a Spectramax M2 plate reader (Molecular Devices Corp., Sunnyvale, CA, USA). Samples were run in duplicate with a repeatability of *r* = 0.82. The results are expressed in units of SOD activity per g homogenate.

#### Total antioxidant capacity: Incubator heat stress

TAC in homogenates of liver, kidney and brain were quantified using a commercially available kit (Antioxidant Assay Kit, Cayman Chemical Co., Ann Arbor, MI, USA) which measures the oxidation of ABTS (2,29-Azino-di-[3-ethybenzthiazoline sulphonate]) by metmyoglobin, which is inhibited by non-enzymatic antioxidants contained in the sample. Oxidized ABTS is measured by spectrophotometry at a wavelength of 750 nm. The capacity of antioxidants in the sample to inhibit oxidation of ABTS is compared with the capacity of known concentrations of Trolox, and the results are expressed as mM of Trolox equivalents per g homogenate. Samples were run in duplicate with a repeatability of *r* = 0.90.

#### Total antioxidant capacity: Simulated heat wave

TAC in homogenates of liver, kidney, and brain were quantified using a commercially available kit (Sigma-Aldrich, cat. no. MAK187 and D2650, MO, USA), following standard techniques [80]. The concentration of large and small molecular antioxidants and total antioxidant capacity can be measured through the conversion of Cu^2+^ ions to Cu^+^, with the reduced Cu^+^ ion chelated with a colourimetric probe read at an absorbance of 570nm. The TAC is compared to an antioxidant activity standard in Trolox equivalents (in 4-20nmole/well). Absorbances were read using an Eon high-performance microplate spectrophotometer (BioTek Instruments Inc., USA). Samples were run in duplicate with a repeatability of *r* = 0.95. Results are expressed as mM of Trolox equivalents per g homogenate.

### Statistical analyses

One control kidney sample was lost for *R*. *dilectus* during the process of analyses for the simulated heatwave experiment. Data were examined for normality and outliers, where outliers were kept to maintain sample size. Normality was tested using the Shapiro-Wilk and Kolmogorov-Smirnov test. Homogeneity of variance was tested using Levene’s test and Brown-Forsythe test. Data were log-transformed where normality was not observed, and we used the appropriate statistical test for unequal variances when homogeneity of variances was not observed. MDA, PC, SOD and TAC levels consisted of data with one independent variable (treatment) separated into two groups (control vs heat stressed). Each tissue was analysed separately. From this, independent samples t-tests were performed to determine the difference in the incubator heat stress and the simulated heat wave treatment from their respective controls. A repeated measures ANOVA was used to determine whether body mass (before and after) significantly changed for each treatment (control and stressed) for each experiment separately (incubator and simulated heat wave). The interactive term body mass x treatment is reported. For significant treatment effects, a partial correlation was performed to determine whether dehydration (determined through changes in body mass before and after an experiment) was significantly correlated to the oxidative marker. Significance was calculated at P<0.05. All analyses were executed using SPSS (version 26) (IBM Corp. Armonk, NY). The results are reported as means ± s.e.

## Results

### Experiment 1: Incubator heat and dehydration stress

**Fig 2:**
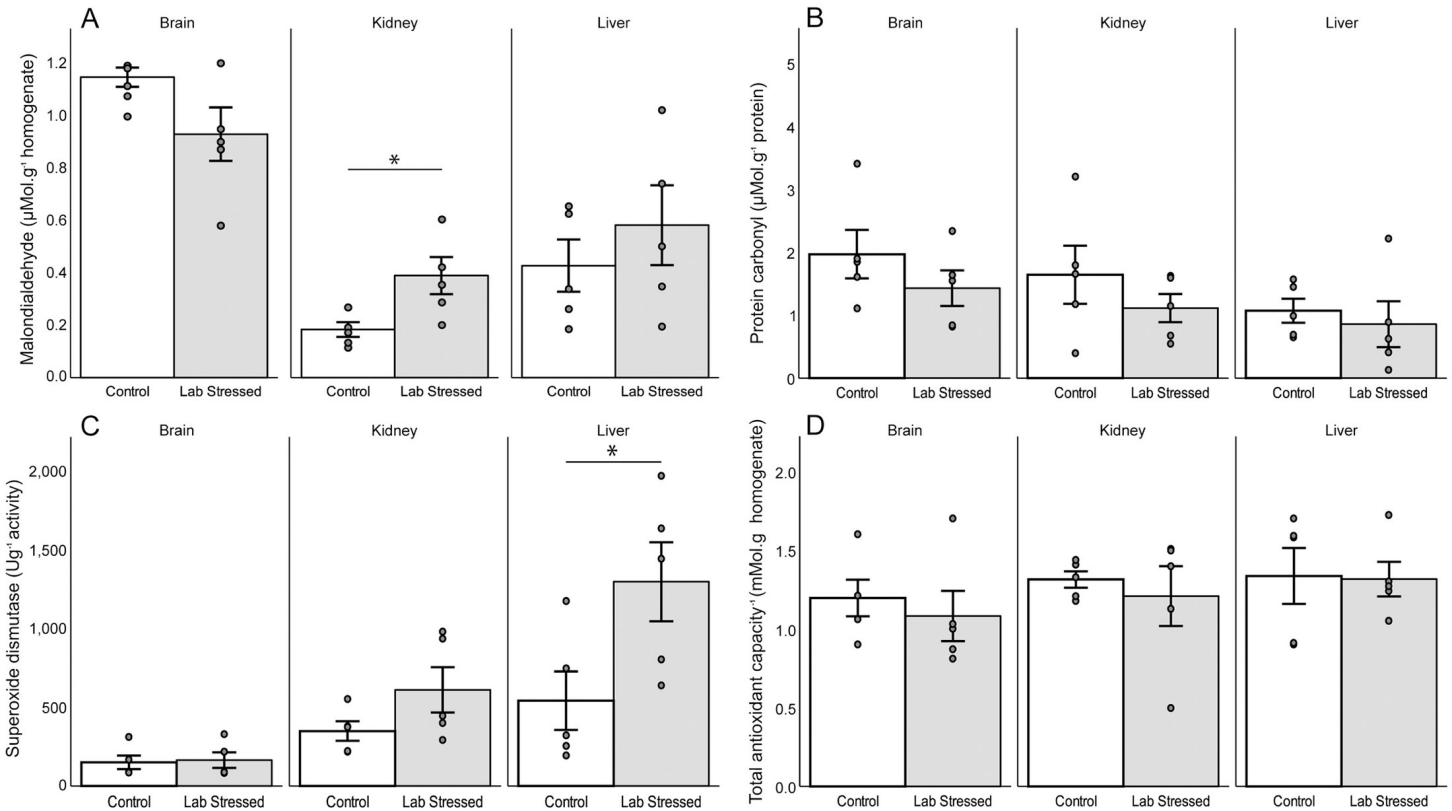
The mean A) malondialdehyde B) protein carbonyl C) superoxide dismutase D) total antioxidant capacity of the brain, kidney and liver in *Rhabdomys dilectus* (N = 5) as a function of an incubator heat stress treatment. Error bars represent ± s.e. Significance at p<0.05.

Lipid peroxidation following the incubator heat stress experiment did not differ significantly from the control in the liver (t-test, t_8_ = 0.85, p = 0.42 or brain (t-test, t_8_ = 2.01, p = 0.10), but was significantly higher in the kidney (t-test, t_8_ = 2.70, p = 0.027) (Fig 2A) compared to the control. Tissues did not significantly differ for protein oxidation following the incubator heat stress experiment when compared with the control (liver: t-test, t_8_ = 0.53, p = 0.61; kidney: t-test, t_8_ = 1.03, p = 0.33; brain: t-test, t_8_ = 1.13, p = 0.29) (Fig 2B). SOD following the incubator heat stress experiment did not significantly differ from the control for the kidney (t-test, t_8_ = 1.69, p = 0.13) or brain (t-test, t_8_ = 0.17, p = 0.87), but was significantly higher in the liver (t-test, t_7.34_ = 2.42, p = 0.045) compared to the control (Fig 2C). TAC following the incubator heat stress experiment did not differ significantly in any tissue (liver: t-test, t_8_ = 0.94, p = 0.93; kidney: t-test, t_8_ = 0.72, p = 0.49; brain: t-test, t_8_ = 0.58, p = 0.58) (Fig 2D). The incubator heat stress group had significantly greater change in body mass4.22 ± 1.79%, compared to the control group, which had 0.45 ± 0.66% change in body mass after the experiment (F_1_ = 23.47, p = 0.001) (S2 Table). Liver SOD activity (N = -0.753, df = 7, p = 0.019) and kidney MDA (r = -0.763, df = 7, p = 0.017) both had a significant negative correlation to body mass after the experiment when controlling for body mass before the experiment (S2 Table).

### Experiment 2: Simulated heat wave

**Fig 3:**
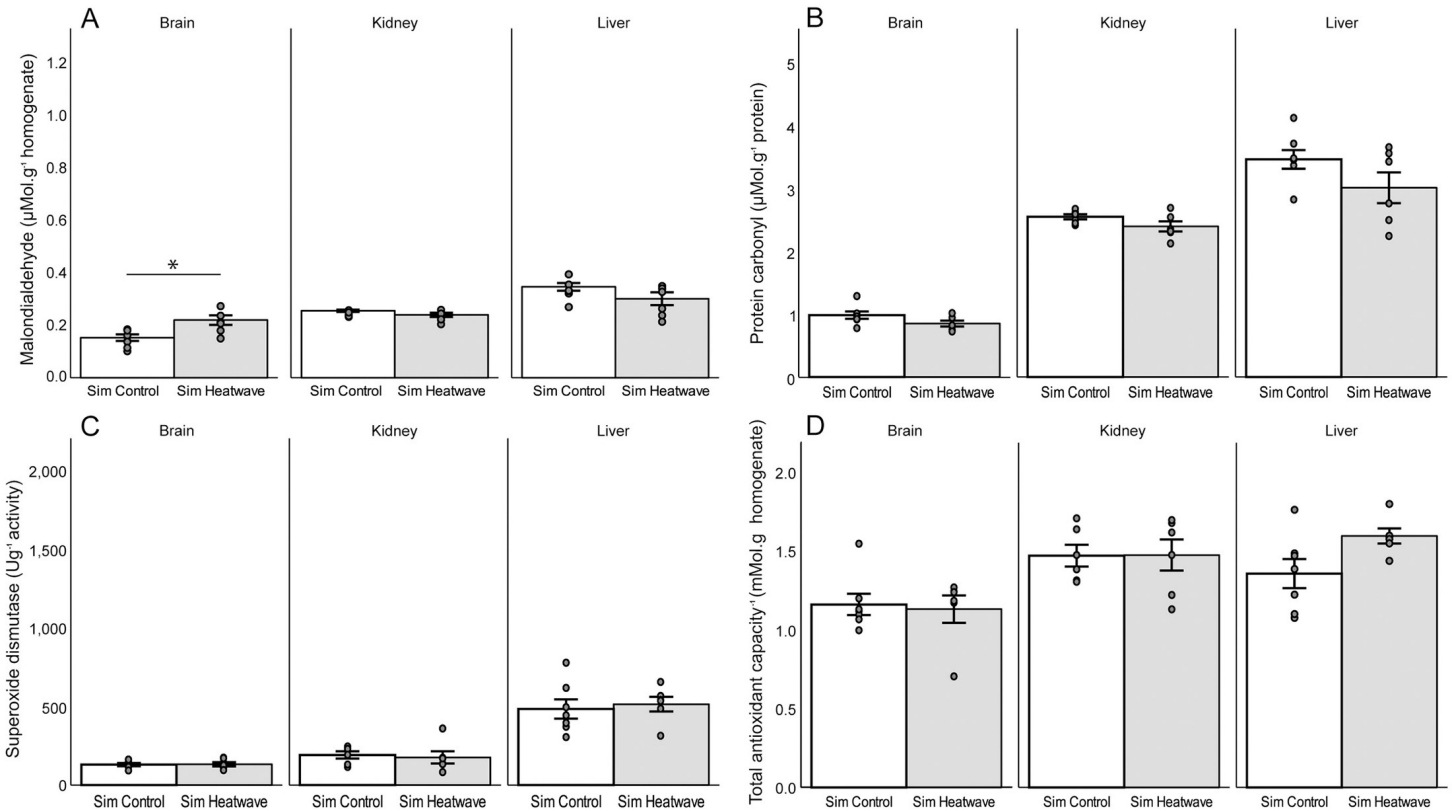
The mean A) malondialdehyde B) protein carbonyl C) superoxide dismutase D) total antioxidant capacity of the brain, kidney and liver *Rhabdomys dilectus* as a function of a simulated heat wave. Control tissues N = 7 (kidney N = 6) and simulated heat wave N = 6. Error bars represent ± s.e. Significance at p<0.05.

Following the simulated heat wave, lipid peroxidation did not significantly differ from the control for the liver (t-test, t_11_ = 0.78, p = 0.45) or kidney (t-test, t_10_ = 0.35, p = 0.74), but was significantly higher for the brain (t-test, t_11_ = 3.10, p = 0.010) (Fig 3A). Protein oxidation following the simulated heat wave did not significantly differ from the control for any of the three tissues (liver: t-test, t_11_ = 1.63, p = 0.13; kidney: t-test, t_10_ = 1.67, p = 0.13; brain: t-test, t_11_ = 1.74, p = 0.11) (Fig 3B). Following the simulated heat wave, SOD did not significantly differ from the control for all tissues (liver: t-test, t_11_ = 0.38, p = 0.71; kidney: t-test, t_10_ = 0.35, p = 0.73; brain: t-test, t_11_ = 0.14, p = 0.89) (Fig 3C). Tissues did not significantly differ from the control in TAC following the simulated heat wave (liver: t-test, t_11_ = 2.18, p = 0.052; kidney: t-test, t_10_ = 0.038, p = 0.97; brain: t-test, t_11_ = 0.27, p = 0.79) (Fig 3D). Both experimental groups had a net positive body mass with no significant difference (F1 = 2.23, p = 0.15) after the experiment, with the control group with 7.96 ± 7.36% change in body mass and the heat stress group had 1.12 ± 7.28% change in body mass after the experiment (S2 Table). No significant partial correlation was observed for brain MDA (p = -0.491, df = 10, p = 0.11) (S2 Table).

## Discussion

Markers of oxidative stress significantly changed in response to two different thermal stress regimes compared to their controls, suggesting thermal and dehydration stress can alter oxidative balance in specific tissues in four striped field mice. Dehydration was determined through the change in body mass, where a body mass exceeding 2% is expected to result in a dehydrated state [81, 82]. The mean from individuals far exceeded this value and it is therefore likely that individuals were dehydrated. In the incubator control group, the mice as a whole demonstrated a net gain in body mass suggesting they were hydrated post-experiment. Body mass measurements were taken prior to the saline injection, which explained the net gain in body mass in the control group while the experimental group lost the weight of the injection and more, resulting in a net body mass loss. Following the incubator heat stress the liver demonstrated antioxidant defense through higher SOD activity preventing significant oxidative damage. In contrast to this, the kidney was susceptible to lipid peroxidation with significantly higher levels of MDA. Interestingly, the liver and kidney did not exhibit any significant changes in oxidative damage or antioxidant defense following the simulated heat wave, with the brain exhibiting significantly higher lipid peroxidation levels.

The magnitude and duration of heat stress strongly affects how tissues will respond [83, 84]. The incubator experiment had a high magnitude and duration of heat stress, which would result in rapid heat gain and high heat loads in tissues [83]. In contrast, in the simulated heat wave, despite a much longer duration to heat stress (3 days), the overall magnitude of heat stress experienced by an animal each day was overall less (±3 hours a day). The incubator heat stress resulted in a net water loss in individuals causing dehydration, whereas sufficient water (food and drinking) during the simulated heat wave allowed individuals to hydrate themselves, possibly resulting in a reduced oxidative stress response due to no net loss of water. Thus susceptibility to heat stress-induced oxidative damage may be very dependent on the duration and magnitude of heat stress experienced, in a tissue-specific manner, as well as highlighting the importance of water availability to circumvent severe dehydration. Heat stress and dehydration together likely results in a compounding effect, and becomes exacerbated depending on the duration and magnitude of the heat stress experienced. This study demonstrated that preventing severe dehydration can minimise the effects of oxidative stress. Previously, dehydration stress has been found to not result in complete recovery after rehydration [41] and future studies may allow for the investigation of oxidative balance in tissues under similar conditions with a recovery period.

Several studies investigating heat induced oxidative stress of the liver have demonstrated elevated lipid peroxidation and reduced SOD activity [68, 85–88]. Heat load (amount of heat absorbed) during heat stress is particularly important in the liver, as the liver has shown a 21 fold increase in heat shock proteins (HSPs) (protective proteins in response to thermal stress) compared to a 12 fold increase in HSPs when the heat load was less [83]. In addition to a higher amount of HSPs produced during high heat loads in the liver, higher heat stress and/or dehydration status have resulted in the rapid upregulation of SOD activity [87, 89–91]. The liver is exposed to hyperosmotic fluids under non-pathogenic conditions and can become hyperosmotic under pathological conditions, which can result in oxidative stress [45, 92, 93]. The present study demonstrated a significant upregulation of SOD activity under heat and dehydration stress, with a finding similar in *Xenopus laevis* (Daudin 1802) where SOD activity increased [94]. Due to SOD activity being significantly increased and not decreased dehydration may have played a larger role in the oxidative stress experienced in the liver. In contrast, since no significant upregulation of antioxidant enzymes were apparent during the simulated heat wave, we believe the heat stress, along with the absence of severe dehydration, was not sufficient to cause any significant oxidative stress in this tissue.

The effect of oxidative damage and antioxidant defense in the kidney in response to disease (e.g. diabetes mellitus) [95, 96], hyperosmolar conditions [45, 97, 98] and HSPs production in response to heat stress has been well documented [83, 99–101]. However, the literature on heat stress influences by itself on the oxidative balance of kidneys is scarce; acute heat stress from exercise incurred no significant oxidative damage in Sprague-Dawley rats [102], but mice exposed to heat stress had higher lipid peroxidation and reduced SOD activity [103]. In broiler chickens, acute heat stress caused a minor decease in SOD activity along with a lower level of lipid peroxidation compared to the control [88]. Goldfish, *Carassius auratus* (Linneaus, 1758) in response to heat stress demonstrated elevated lipid peroxidation, minimal changes in SOD activity, but increased in glutathione enzymes, which demonstrated higher expression of other antioxidant enzymes in response to heat stress. Kidneys are sensitive to oxidative damage [104, 105], which is caused by hyperosmolality, which is more likely under the duress of increased temperatures resulting in dehydration [98]. In this study, the kidneys likely became heat stressed and dehydrated (observed from the % body mass deficit), which may have resulted in hyperosmolality [98, 106]. Hypersomolality in the kidneys activate the polyol-fructokinase pathway and possibly the chronic effects of vasopressin to induce tubular and glomerular injury, both which cause oxidative damage [98]. The importance of kidney hydrative state is emphasized in this study, as the limited water availability, along with dehydration following the incubator experiment was associated with significantly increased oxidative stress. This may also explain why animals may have become active during a simulated heat wave to drink water to prevent dehydration [19], and in turn would prevent kidney oxidative stress. Overall, kidney oxidative stress may be very reliant on the urinary concentrating ability of the animals resulting in different tolerances to hyperosmolality, with desert animals better equipped with kidney oxidative stress [107–109].

The brain is known for its susceptibility to heat stroke resulting in multiple organ dysfunction [63, 110, 111]. The brain in laboratory mice undergoing acute heat stress from exercise demonstrated decreases in MDA and PC [102], however, during acute heat stress at extreme temperatures, brain SOD activity decreased accompanied by lipid peroxidation increases [62, 111, 112]. In contrast, the simulated heat wave demonstrated increased lipid peroxidation, but no changes in antioxidant enzyme activity [62, 111, 112]. Despite the magnitude and duration of heat stress in the incubator experiment, the brain did not significantly increase in oxidative damage. The duration of heat stress following the simulated heat wave was much longer and may have compromised the permeability of the blood-brain barrier (BBB) and resulted in a cascading effect of increased oxidative damage [113, 114]. Due to dehydration having a minimal effect following the simulated heat wave, it was likely that heat stress alone was sufficient to cause oxidative stress in the brain over long time periods, with more severe temperature exposures likely to be more deleterious [115].

In light of climate change, currently for a mesic crepuscular rodent the four striped field mouse, a three-day heat wave with freely available water is enough to induce oxidative damage in the brain, when measured in the absence of behavioural and physiological thermoregulation (e.g. microsites). In addition to current conditions (assuming no behavioural and physiological thermoregulation), whole-body fever range temperatures and dehydration may likely result in kidney and liver oxidative stress. The kidney will suffer oxidative damage when dehydrated and highlights the importance of water to animals during a heat stress to offset dehydration and to potentially rehydrate [19]. It is, however, uncertain to what extent oxidative damage repair mechanisms may reduce potential sub-lethal consequences from heat induced oxidative stress [41, 116–120]. Differences observed between the tissues demonstrates the importance of assessing oxidative damage and antioxidant defenses in different tissues to obtain an overview of an organism’s physiological state [61].

## Supporting information

S1 Table The control and stressed PIT animal body temperature recordings inside the incubator throughout the 6 hour period. (XLS)

S2 Table The body mass before, body mass after and % body mass change of each individual across all experiments. (XLSX)

## References

[1] Robinson PJ. On the definition of a heat wave. Journal of applied Meteorology. 2001;40(4):762–75.

[2] Souch C, Grimmond C. Applied Climatology:‘heat waves’. Progress in Physical Geography. 2004;28(4):599–606.

[3] Conradie SR, Woodborne SM, Cunningham SJ, McKechnie AE. Chronic, sublethal effects of high temperatures will cause severe declines in southern African arid-zone birds during the 21st century. Proceedings of the National Academy of Sciences. 2019:201821312 10.1073/pnas.1821312116 31235571 PMC6628835

[4] McKechnie AE, Wolf BO. Climate change increases the likelihood of catastrophic avian mortality events during extreme heat waves. Biology letters. 2010;6(2):253–6. 10.1098/rsbl.2009.0702 19793742 PMC2865035

[5] Lovegrove BG, Canale C, Levesque D, Fluch G, Řeháková-Petrů M, Ruf T. Are tropical small mammals physiologically vulnerable to Arrhenius effects and climate change? Physiological and Biochemical Zoology. 2013;87(1):30–45. 10.1086/673313 24457919

[6] Saunders DA, Mawson P, Dawson R. The impact of two extreme weather events and other causes of death on Carnaby’s black cockatoo: a promise of things to come for a threatened species? Pacific Conservation Biology. 2011;17(2):141–8.

[7] Easterling DR, Meehl GA, Parmesan C, Changnon SA, Karl TR, Mearns LO. Climate extremes: observations, modeling, and impacts. science. 2000;289(5487):2068–74. 10.1126/science.289.5487.2068 11000103

[8] Meehl GA, Tebaldi C. More intense, more frequent, and longer lasting heat waves in the 21st century. Science. 2004;305(5686):994–7. 10.1126/science.1098704 15310900

[9] Poumadere M, Mays C, Le Mer S, Blong R. The 2003 heat wave in France: dangerous climate change here and now. Risk Analysis: an International Journal. 2005;25(6):1483–94. 10.1111/j.1539-6924.2005.00694.x 16506977

[10] IPCC. Climate change 2014: impacts, adaptation, and vulnerability. Part A: global and sectoral aspects. In C.B. Field, V.R. Barros, D.J. Dokken, K.J. Mach, M.D. Mastrandrea, T.E. Bilir, M. Chatterjee, et al., eds. Contribution of Working Group II to the fifth assessment report of the Intergovernmental Panel on Climate Change: Cambridge University Press, Cambridge; 2014.

[11] Vasseur DA, DeLong JP, Gilbert B, Greig HS, Harley CD, McCann KS, et al Increased temperature variation poses a greater risk to species than climate warming. Proceedings of the Royal Society B: Biological Sciences. 2014;281(1779):20132612 10.1098/rspb.2013.2612 24478296 PMC3924069

[12] Luber G, McGeehin M. Climate change and extreme heat events. American journal of preventive medicine. 2008;35(5):429–35. 10.1016/j.amepre.2008.08.021 18929969

[13] Thomas CD, Cameron A, Green RE, Bakkenes M, Beaumont LJ, Collingham YC, et al Extinction risk from climate change. Nature. 2004;427(6970):145–8. 10.1038/nature02121 14712274

[14] Scheffers BR, Edwards DP, Diesmos A, Williams SE, Evans TA. Microhabitats reduce animal’s exposure to climate extremes. Global change biology. 2014;20(2):495–503. 10.1111/gcb.12439 24132984

[15] Bozinovic F, Lagos JA, Vásquez RA, Kenagy G. Time and energy use under thermoregulatory constraints in a diurnal rodent. J Therm Biol. 2000;25(3):251–6.

[16] Mitchell D, Snelling EP, Hetem RS, Maloney SK, Strauss WM, Fuller A. Revisiting concepts of thermal physiology: predicting responses of mammals to climate change. J Anim Ecol. 2018 10.1111/1365-2656.12818 29479693

[17] Maclean I, editor Adapting nature conservation to climate change: the importance of microclimate. ECCB2018: 5th European Congress of Conservation Biology 12th-15th of June 2018, Jyväskylä, Finland; 2018: Open Science Centre, University of Jyväskylä.

[18] Gardner JL, Peters A, Kearney MR, Joseph L, Heinsohn R. Declining body size: a third universal response to warming? Trends in ecology & evolution. 2011;26(6):285–91. 10.1016/j.tree.2011.03.005 21470708

[19] Jacobs PJ, Bennett NC, Oosthuizen MK. Locomotor activity in field captured crepuscular four-striped field mice, Rhabdomys dilectus and nocturnal Namaqua rock mice, Micaelamys namaquensis during a simulated heat wave. Journal of Thermal Biology. 2020;87:102479 10.1016/j.jtherbio.2019.102479 32001021

[20] Hainsworth F. Saliva spreading, activity, and body temperature regulation in the rat. American Journal of Physiology-Legacy Content. 1967;212(6):1288–92. 10.1152/ajplegacy.1967.212.6.1288 4952116

[21] Hainsworth F, Stricker EM, Epstein A. Water metabolism of rats in the heat: dehydration and drinking. American Journal of Physiology-Legacy Content. 1968;214(5):983–9. 10.1152/ajplegacy.1968.214.5.983 5647202

[22] Sassi PL, Novillo A. Acclimating to thermal changes: Intraspecific variation in a small mammal from the Andes Mountains. Mammalian Biology-Zeitschrift für Säugetierkunde. 2015;80(2):81–6.

[23] Hammond KA, Szewczak J, Król E. Effects of altitude and temperature on organ phenotypic plasticity along an altitudinal gradient. Journal of Experimental Biology. 2001;204(11):1991–2000. 11441040 10.1242/jeb.204.11.1991

[24] Zub K, Szafrańska P, Konarzewski M, Redman P, Speakman J. Trade-offs between activity and thermoregulation in a small carnivore, the least weasel Mustela nivalis. Proceedings of the Royal Society of London B: Biological Sciences. 2009:rspb. 2008.1936. 10.1098/rspb.2008.1936 PMC2674502 19324766

[25] Murray IW, Smith FA. Estimating the influence of the thermal environment on activity patterns of the desert woodrat (Neotoma lepida) using temperature chronologies. Can J Zool. 2012;90(9):1171–80.

[26] Withers PC, Cooper CE, Maloney SK, Bozinovic F, Cruz-Neto AP. Ecological and environmental physiology of mammals: Oxford University Press; 2016.

[27] Folk GE, Semken A. The evolution of sweat glands. International Journal of Biometeorology. 1991;35(3):180–6. 10.1007/BF01049065 1778649

[28] McKechnie AE, Wolf BO. The physiology of heat tolerance in small endotherms. Physiology. 2019;34(5):302–13. 10.1152/physiol.00011.2019 31389778

[29] Rymer T, Pillay N, Schradin C. Extinction or survival? Behavioral flexibility in response to environmental change in the African striped mouse *Rhabdomys*. Sustainability. 2013;5(1):163–86.

[30] Du Toit N, Pillay N., Ganem G. & Relton C. Rhabdomys dilectus The IUCN Red List of Threatened Species 2019;(e.T112168645A140971990.). 10.2305/IUCN.UK.2019-1.RLTS.T112168645A140971990.en

[31] Haim A, Fourie FlR. Heat production in nocturnal (Praomys natalensis) and diurnal (Rhabdomys pumilio) South African murids. South African Journal of Zoology. 1980;15(2):91–4.

[32] Birben E, Sahiner UM, Sackesen C, Erzurum S, Kalayci O. Oxidative stress and antioxidant defense. World Allergy Organization Journal. 2012;5(1):9 23268465 10.1097/WOX.0b013e3182439613 PMC3488923

[33] Halliwell B. Biochemistry of oxidative stress. Portland Press Limited; 2007. 10.1042/BST0351147 17956298

[34] Droge W. Free radicals in the physiological control of cell function. Physiological reviews. 2002;82(1):47–95. 10.1152/physrev.00018.2001 11773609

[35] Giordano FJ. Oxygen, oxidative stress, hypoxia, and heart failure. The Journal of clinical investigation. 2005;115(3):500–8. 10.1172/JCI24408 15765131 PMC1052012

[36] Blair AS, Hajduch E, Litherland GJ, Hundal HS. Regulation of Glucose Transport and Glycogen Synthesis in L6 Muscle Cells during Oxidative stress evidence for cross-talk between the insulin and sapk2/p38 mitogen-activated protein kinase signaling pathways. Journal of Biological Chemistry. 1999;274(51):36293–9. 10.1074/jbc.274.51.36293 10593919

[37] Sies H. Oxidative stress: a concept in redox biology and medicine. Redox biology. 2015;4:180–3. 10.1016/j.redox.2015.01.002 25588755 PMC4309861

[38] Oliveira MF, Geihs MA, França TF, Moreira DC, Hermes-Lima M. Is “preparation for oxidative stress” a case of physiological conditioning hormesis? Frontiers in physiology. 2018;9:945 10.3389/fphys.2018.00945 30116197 PMC6082956

[39] Burg MB, Ferraris JD, Dmitrieva NI. Cellular response to hyperosmotic stresses. Physiological reviews. 2007;87(4):1441–74. 10.1152/physrev.00056.2006 17928589

[40] Hillman AR, Vince RV, Taylor L, McNaughton L, Mitchell N, Siegler J. Exercise-induced dehydration with and without environmental heat stress results in increased oxidative stress. Appl Physiol Nutr Metab. 2011;36(5):698–706. 10.1139/h11-080 21980993

[41] Georgescu VP, de Souza TP Junior, Behrens C, Barros MP, Bueno CA, Utter AC, et al Effect of exercise-induced dehydration on circulatory markers of oxidative damage and antioxidant capacity. Appl Physiol Nutr Metab. 2017;42(7):694–9. 10.1139/apnm-2016-0701 28182858

[42] Laitano O, Kalsi KK, Pook M, Oliveira AR, González-Alonso J. Separate and combined effects of heat stress and exercise on circulatory markers of oxidative stress in euhydrated humans. Eur J Appl Physiol. 2010;110(5):953–60. 10.1007/s00421-010-1577-5 20658249

[43] Slimen IB, Najar T, Ghram A, Dabbebi H, Ben Mrad M, Abdrabbah M. Reactive oxygen species, heat stress and oxidative-induced mitochondrial damage. A review. International Journal of Hyperthermia. 2014;30(7):513–23. 10.3109/02656736.2014.971446 25354680

[44] Habashy WS, Milfort MC, Rekaya R, Aggrey SE. Cellular antioxidant enzyme activity and biomarkers for oxidative stress are affected by heat stress. International journal of biometeorology. 2019;63(12):1569–84. 10.1007/s00484-019-01769-z 31352522

[45] Schliess F, Häussinger D. The cellular hydration state: a critical determinant for cell death and survival. Biol Chem. 2002;383(3–4):577–83. 10.1515/BC.2002.059 12033446

[46] França M, Panek A, Eleutherio E. Oxidative stress and its effects during dehydration. Comparative Biochemistry and Physiology Part A: Molecular & Integrative Physiology. 2007;146(4):621–31. 10.1016/j.cbpa.2006.02.030 16580854

[47] Navari-Izzo F, Quartacci M, Sgherri C. Superoxide generation in relation to dehydration and rehydration. Portland Press Ltd; 1996 10.1042/bst0240447 8736781

[48] Richter C, Park J-W, Ames BN. Normal oxidative damage to mitochondrial and nuclear DNA is extensive. Proceedings of the National Academy of Sciences. 1988;85(17):6465–7. 10.1073/pnas.85.17.6465 PMC281993 3413108

[49] LeDoux SP, Driggers WJ, Hollensworth BS, Wilson GL. Repair of alkylation and oxidative damage in mitochondrial DNA. Mutation Research. 1999;434(3):149–59. 10.1016/s0921-8777(99)00026-9 10486589

[50] Rubbo H, Radi R, Trujillo M, Telleri R, Kalyanaraman B, Barnes S, et al Nitric oxide regulation of superoxide and peroxynitrite-dependent lipid peroxidation. Formation of novel nitrogen-containing oxidized lipid derivatives. Journal of Biological Chemistry. 1994;269(42):26066–75. 7929318

[51] Standman E, Levine R. Protein Oxidation. Annals of the New York Academy of Sciences. 2000;899:191–208. 10.1111/j.1749-6632.2000.tb06187.x 10863540

[52] Kalmar B, Greensmith L. Induction of heat shock proteins for protection against oxidative stress. Advanced Drug Delivery Reviews. 2009;61(4):310–8. 10.1016/j.addr.2009.02.003 19248813

[53] Forster MJ, Dubey A, Dawson KM, Stutts WA, Lal H, Sohal RS. Age-related losses of cognitive function and motor skills in mice are associated with oxidative protein damage in the brain. Proceedings of the National Academy of Sciences. 1996;93(10):4765–9. 10.1073/pnas.93.10.4765 PMC39353 8643477

[54] Paul C, Teng S, Saunders PT. A single, mild, transient scrotal heat stress causes hypoxia and oxidative stress in mouse testes, which induces germ cell death. Biology of Reproduction. 2009;80(5):913–9. 10.1095/biolreprod.108.071779 19144962 PMC2709966

[55] Tvrdá E, Kňažická Z, Bárdos L, Massányi P, Lukáč N. Impact of oxidative stress on male fertility—A review. Acta veterinaria hungarica. 2011;59(4):465–84. 22079708 10.1556/AVet.2011.034

[56] Bize P, Criscuolo F, Metcalfe NB, Nasir L, Monaghan P. Telomere dynamics rather than age predict life expectancy in the wild. Proceedings of the Royal Society B: Biological Sciences. 2009;276(1662):1679–83. 10.1098/rspb.2008.1817 PMC2660992 19324831

[57] von Zglinicki T. Oxidative stress shortens telomeres. Trends in biochemical sciences. 2002;27(7):339–44. 10.1016/s0968-0004(02)02110-2 12114022

[58] Rodríguez-Estival J, García-de Blas E, Smits JE. Oxidative stress biomarkers indicate sublethal health effects in a sentinel small mammal species, the deer mouse (Peromyscus maniculatus), on reclaimed oil sands areas. Ecological Indicators. 2016;62:66–75.

[59] Stahlschmidt Z, French S, Ahn A, Webb A, Butler MW. A simulated heat wave has diverse effects on immune function and oxidative physiology in the corn snake (*Pantherophis guttatus*). Physiol Biochem Zool. 2017;90(4):434–44. 28398156 10.1086/691315

[60] Wang Z, O’Connor TP, Heshka S, Heymsfield SB. The reconstruction of Kleiber’s law at the organ-tissue level. The journal of nutrition. 2001;131(11):2967–70. 10.1093/jn/131.11.2967 11694627

[61] Schmidt CM, Blount JD, Bennett NC. Reproduction is associated with a tissue-dependent reduction of oxidative stress in eusocial female Damaraland mole-rats (Fukomys damarensis). PloS one. 2014;9(7):e103286. 25068591 10.1371/journal.pone.0103286 PMC4113376

[62] Hsu S-F, Niu K-C, Lin C-L, Lin M-T. Brain cooling causes attenuation of cerebral oxidative stress, systemic inflammation, activated coagulation, and tissue ischemia/injury during heatstroke. Shock. 2006;26(2):210–20. 16878031 10.1097/01.shk.0000223124.49265.10

[63] Chen S-H, Lin M-T, Chang C-P. Ischemic and oxidative damage to the hypothalamus may be responsible for heat stroke. Current Neuropharmacology. 2013;11(2):129–40. 23997749 10.2174/1570159X11311020001 PMC3637668

[64] Balat A, Resic H, Bellinghieri G, Anarat A. Devil’s Triangle in Kidney Diseases: Oxidative Stress, Mediators, and Inflammation. International journal of nephrology. 2012;2012. 10.1155/2012/156286 PMC3529894 23304502

[65] Ayala A, Muñoz MF, Argüelles S. Lipid peroxidation: production, metabolism, and signaling mechanisms of malondialdehyde and 4-hydroxy-2-nonenal. Oxidative Medicine and Cellular Longevity. 2014;2014:1–31. 10.1155/2014/360438 PMC4066722 24999379

[66] Bharti VK, Srivastava R, Kumar H, Bag S, Majumdar A, Singh G, et al Effects of melatonin and epiphyseal proteins on fluoride-induced adverse changes in antioxidant status of heart, liver, and kidney of rats. Advances in pharmacological sciences. 2014;2014. 10.1155/2014/532969 PMC3984810 24790596

[67] Balcerczyk A, Bartosz G. Thiols are main determinants of total antioxidant capacity of cellular homogenates. Free radical research. 2003;37(5):537–41. 10.1080/1071576031000083189 12797475

[68] Zhang HJ, Doctrow SR, Xu L, Oberley LW, Beecher B, Morrison J, et al Redox modulation of the liver with chronic antioxidant enzyme mimetic treatment prevents age-related oxidative damage associated with environmental stress. The FASEB journal. 2004;18(13):1547–9. 15319374 10.1096/fj.04-1629fje

[69] Zhang Z, Ferraris JD, Brooks HL, Brisc I, Burg MB. Expression of osmotic stress-related genesin tissues of normal and hypoosmotic rats. American Journal of Physiology-Renal Physiology. 2003;4(285):F688–F93. 10.1152/ajprenal.00028.2003 12824075

[70] Bloomer SA, Kregel KC, Brown KE. Heat stress stimulates hepcidin mRNA expression and C/EBPα protein expression in aged rodent liver. Archives of gerontology and geriatrics. 2014;58(1):145–52. 10.1016/j.archger.2013.07.012 23993269 PMC3808518

[71] Tiidus PM. Estrogen and gender effects on muscle damage, inflammation, and oxidative stress. Canadian Journal of Applied Physiology. 2000;25(4):274–87. 10.1139/h00-022 10953066

[72] Miller AA, De Silva TM, Jackman KA, Sobey CG. Effect of gender and sex hormones on vascular oxidative stress. Clinical and Experimental Pharmacology and Physiology. 2007;34(10):1037–43. 10.1111/j.1440-1681.2007.04732.x 17714091

[73] Katalinic V, Modun D, Music I, Boban M. Gender differences in antioxidant capacity of rat tissues determined by 2, 2′-azinobis (3-ethylbenzothiazoline 6-sulfonate; ABTS) and ferric reducing antioxidant power (FRAP) assays. Comparative Biochemistry and Physiology Part C: Toxicology & Pharmacology. 2005;140(1):47–52. 10.1016/j.cca.2005.01.005 15792622

[74] Ostberg J, Kaplan K, Repasky E. Induction of stress proteins in a panel of mouse tissues by fever-range whole body hyperthermia. International journal of hyperthermia. 2002;18(6):552–62. 10.1080/02656730210166168 12537754

[75] Halliwell B, Chirico S. Lipid peroxidation: its mechanism, measurement, and significance. The American journal of clinical nutrition. 1993;57(5):715S–25S. 8475889 10.1093/ajcn/57.5.715S

[76] Dalle-Donne I, Rossi R, Giustarini D, Milzani A, Colombo R. Protein carbonyl groups as biomarkers of oxidative stress. Clinica Chimica Acta. 2003;329(1–2):23–38. 10.1016/s0009-8981(03)00003-2 12589963

[77] Levine RL, Garland D, Oliver CN, Amici A, Climent I, Lenz A-G, et al Determination of carbonyl content in oxidatively modified proteins. Methods Enzymol. 186: Elsevier; 1990 p. 464–78. 10.1016/0076-6879(90)86141-h 1978225

[78] Naito Y, Masaichi C-iL, Kato Y, Nagai R, Yonei Y. Oxidative stress markers. Anti-Aging Medicine. 2010;7(5):36–44.

[79] Spitz DR, Oberley LW. An assay for superoxide dismutase activity in mammalian tissue homogenates. Analytical biochemistry. 1989;179(1):8–18. 10.1016/0003-2697(89)90192-9 2547324

[80] Erel O. A novel automated method to measure total antioxidant response against potent free radical reactions. Clinical biochemistry. 2004;37(2):112–9. 10.1016/j.clinbiochem.2003.10.014 14725941

[81] Kenefick RW, Cheuvront SN. Physiological adjustments to hypohydration: Impact on thermoregulation. Autonomic Neuroscience. 2016;196:47–51. 10.1016/j.autneu.2016.02.003 26944095

[82] Cheuvront SN, Haymes EM. Thermoregulation and marathon running. Sports Med. 2001;31(10):743–62. 10.2165/00007256-200131100-00004 11547895

[83] Flanagan S, Ryan A, Gisolfi C, Moseley P. Tissue-specific HSP70 response in animals undergoing heat stress. American Journal of Physiology-Regulatory, Integrative and Comparative Physiology. 1995;268(1):R28–R32. 10.1152/ajpregu.1995.268.1.R28 7840333

[84] Mizzen LA, Welch WJ. Characterization of the thermotolerant cell. I. Effects on protein synthesis activity and the regulation of heat-shock protein 70 expression. The Journal of cell biology. 1988;106(4):1105–16. 10.1083/jcb.106.4.1105 3360849 PMC2114998

[85] Şahin E, Gümüşlü S. Immobilization stress in rat tissues: alterations in protein oxidation, lipid peroxidation and antioxidant defense system. Comparative Biochemistry and Physiology Part C: Toxicology & Pharmacology. 2007;144(4):342–7. 10.1016/j.cbpc.2006.10.009 17157074

[86] Das A. Heat stress-induced hepatotoxicity and its prevention by resveratrol in rats. Toxicology mechanisms and methods. 2011;21(5):393–9. 10.3109/15376516.2010.550016 21426263

[87] Lin H, Decuypere E, Buyse J. Acute heat stress induces oxidative stress in broiler chickens. Comparative Biochemistry and Physiology Part A: Molecular & Integrative Physiology. 2006;144(1):11–7. 10.1016/j.cbpa.2006.01.032 16517194

[88] Lin H, Du R, Zhang Z. Peroxide status in tissues of heat-stressed broilers. Asian-Australasian Journal of Animal Sciences. 2000;13(10):1373–6.

[89] Altan Ö, Pabuçcuoğlu A, Altan A, Konyalioğlu S, Bayraktar H. Effect of heat stress on oxidative stress, lipid peroxidation and some stress parameters in broilers. British Poultry Science. 2003;44(4):545–50. 10.1080/00071660310001618334 14584844

[90] Öztürk O, Gümüşlü S. Age-related changes of antioxidant enzyme activities, glutathione status and lipid peroxidation in rat erythrocytes after heat stress. Life sciences. 2004;75(13):1551–65. 10.1016/j.lfs.2004.03.020 15261761

[91] Skibba J, Gwartney E. Liver hyperthermia and oxidative stress: role of iron and aldehyde production. International journal of hyperthermia. 1997;13(2):215–26. 10.3109/02656739709012384 9147147

[92] Brocker C, Thompson DC, Vasiliou V. The role of hyperosmotic stress in inflammation and disease. Biomolecular concepts. 2012;3(4):345–64. 10.1515/bmc-2012-0001 22977648 PMC3438915

[93] Aramburu J, López-Rodríguez C. Brx shines a light on the route from hyperosmolarity to NFAT5. Science Signaling. 2009;2(65):pe20–pe. 10.1126/scisignal.265pe20 19351952

[94] Malik AI, Storey KB. Transcriptional regulation of antioxidant enzymes by FoxO1 under dehydration stress. Gene. 2011;485(2):114–9. 10.1016/j.gene.2011.06.014 21708231

[95] Ozbek E. Induction of oxidative stress in kidney. International journal of nephrology. 2012;2012 10.1155/2012/465897 22577546 PMC3345218

[96] Asmat U, Abad K, Ismail K. Diabetes mellitus and oxidative stress—A concise review. Saudi Pharmaceutical Journal. 2016;24(5):547–53. 10.1016/j.jsps.2015.03.013 27752226 PMC5059829

[97] Rosas-Rodríguez JA, Valenzuela-Soto EM. Enzymes involved in osmolyte synthesis: How does oxidative stress affect osmoregulation in renal cells? Life sciences. 2010;87(17–18):515–20. 10.1016/j.lfs.2010.08.003 20727361

[98] Johnson RJ, Sánchez-Lozada LG, Newman LS, Lanaspa MA, Diaz HF, Lemery J, et al Climate change and the kidney. Annals of Nutrition and Metabolism. 2019;74(3):38–44. 31203298 10.1159/000500344

[99] Yu BP. Cellular defenses against damage from reactive oxygen species. Physiological Reviews. 1994;74(1):139–62. 10.1152/physrev.1994.74.1.139 8295932

[100] Sreedhar A, Pardhasaradhi B, Khar A, Srinivas UK. A cross talk between cellular signalling and cellular redox state during heat-induced apoptosis in a rat histiocytoma. Free Radical Biology and Medicine. 2002;32(3):221–7. 10.1016/s0891-5849(01)00796-1 11827747

[101] Lovis C, Mach F, Donati YR, Bonventre JV, Polla BS. Heat shock proteins and the kidney. Renal failure. 1994;16(2):179–92. 10.3109/08860229409044859 8041958

[102] Liu J, Yeo HC, Overvik-Douki E, Hagen T, Doniger SJ, Chu DW, et al Chronically and acutely exercised rats: biomarkers of oxidative stress and endogenous antioxidants. Journal of Applied Physiology. 2000;89(1):21–8. 10.1152/jappl.2000.89.1.21 10904031

[103] Peng X-y, Ji L-p. Effects of Free Radical Metabolism in Kidney of Mice after Heat Stress [J]. Yinshan Academic Journal (Natural Science Edition). 2009;3.

[104] Halliwell B, Gutteridge J. *Free radical in biology and medicine*: 2nd ed UK: Clarendon: Oxford Science Publications; 1989.

[105] Hulbert AJ, Turner N, Hinde J, Else P, Guderley H. How might you compare mitochondria from different tissues and different species? Journal of Comparative Physiology B. 2006;176(2):93–105. 10.1007/s00360-005-0025-z 16408229

[106] Kültz D. Hyperosmolality triggers oxidative damage in kidney cells. Proceedings of the National Academy of Sciences. 2004;101(25):9177–8. 10.1073/pnas.0403241101 15199186 PMC438948

[107] Segar WE. Chronic Hyperosmolality: A Condition Resulting From Absence of Thirst, Defective Osmoregulation, and Limited Ability to Concentrate Urine. American Journal of Diseases of Children. 1966;112(4):318–27. 5947981 10.1001/archpedi.1966.02090130092008

[108] Schmidt-Nielsen K, Schmidt-Nielsen B, Schneiderman H. Salt excretion in desert mammals. American Journal of Physiology-Legacy Content. 1948;154(1):163–6. 10.1152/ajplegacy.1948.154.1.163 18893073

[109] Donald J, Pannabecker TL. Osmoregulation in desert-adapted mammals Sodium and Water Homeostasis: Springer; 2015 p. 191–211.

[110] Hubbard R, Bowers W, Matthew W, Curtis F, Criss R, Sheldon G, et al Rat model of acute heatstroke mortality. Journal of Applied Physiology. 1977;42(6):809–16. 10.1152/jappl.1977.42.6.809 881380

[111] Yang C-Y, Lin M-T. Oxidative stress in rats with heatstroke-induced cerebral ischemia. Stroke. 2002;33(3):790–4. 11872905 10.1161/hs0102.100208

[112] Chang C-K, Chang C-P, Liu S-Y, Lin M-T. Oxidative stress and ischemic injuries in heat stroke. Progress in brain research. 2007;162:525–46. 10.1016/S0079-6123(06)62025-6 17645935

[113] Sharma H, Duncan J, Johanson C. Whole-body hyperthermia in the rat disrupts the blood-cerebrospinal fluid barrier and induces brain edema Brain Edema XIII: Springer; 2006 p. 426–31. 10.1007/3-211-30714-1_88 16671499

[114] Lochhead JJ, McCaffrey G, Quigley CE, Finch J, DeMarco KM, Nametz N, et al Oxidative stress increases blood–brain barrier permeability and induces alterations in occludin during hypoxia–reoxygenation. Journal of Cerebral Blood Flow & Metabolism. 2010;30(9):1625–36. 20234382 10.1038/jcbfm.2010.29 PMC2949263

[115] Sharma H. Heat-related deaths are largely due to brain damage. Indian Journal of Medical Research. 2005;121(5):621 15937362

[116] Shacter E. Quantification and significance of protein oxidation in biological samples. Drug metabolism reviews. 2000;32(3–4):307–26. 10.1081/dmr-100102336 11139131

[117] Wang Z, Rhee DB, Lu J, Bohr CT, Zhou F, Vallabhaneni H, et al Characterization of oxidative guanine damage and repair in mammalian telomeres. PLoS genetics. 2010;6(5). 10.1371/journal.pgen.1000951 20485567 PMC2869316

[118] Nussey DH, Pemberton JM, Pilkington JG, Blount JD. Life history correlates of oxidative damage in a free‐living mammal population. Functional Ecology. 2009;23(4):809–17.

[119] Beckman KB, Ames BN. The free radical theory of aging matures. Physiological reviews. 1998;78(2):547–81. 10.1152/physrev.1998.78.2.547 9562038

[120] Li H, Benipal B, Zhou S, Dodia C, Chatterjee S, Tao J-Q, et al Critical role of peroxiredoxin 6 in the repair of peroxidized cell membranes following oxidative stress. Free Radical Biology and Medicine. 2015;87:356–65. 10.1016/j.freeradbiomed.2015.06.009 26117327 PMC4780751

