# Supplementary material for: Heat and dehydration induced oxidative damage and antioxidant defenses following incubator heat stress and a simulated heat wave in wild caught four-striped field mice Rhabdomys dilectus
Source: PLoS One. 2020 Nov 13;15(11):e0242279. doi: 10.1371/journal.pone.0242279 (PMC7665817; doi:10.1371/journal.pone.0242279)
Supplement: Attachment — Submitted filename: Review PONE-D-20-14410.pdf [file pone.0242279.s003.pdf]

I felt very excited when I received the invitation to be a reviewer of this manuscript. Despite a little bit long, the title of the paper gave me a lot of expectations about an integrative ecophysiology study that would mix a least two different levels of organization: 1. the cellular, analyzing changes in the oxidative balance, and 2. the organismal, associating thermal biology with dehydration. Unfortunately, this expectation has not been totally covered across the manuscript. In my opinion, the description of the methods and results related to the redox balance of the animals are good, despite the bad presentation of table 1 and the fact that the authors just test one antioxidative enzyme (SOD). On the other hand, where I found more weaknesses were in the link between the result of the oxidative stress of the individuals and the data of body temperature and its association with the dehydration of the animals (if exist and/or if it is significant for the oxidative stress process).

To begin, I did not find the data of body temperature that the authors reported in the section of materials and methods (measured with a temperature-sensitive PIT). These data are the backbone that supports all the thermal biology analyses, critically required to understand the impact of the incubator heat stress in the animals and the simulated heat wave experiments. Likewise, the authors do not show an analysis about the dehydration process between the treatments (and/or among the individuals of the same treatment), which is a great error because one of their main conclusion is related to a critical role of magnitude, duration and water availability for the stress experienced by each tissue and its influences in redox balance of each one of them. Further comments on the manuscript are presented in the following section. I can see great potential in this study, and I suggest the authors to organize more the result presentation to get the link between thermal biology (dehydration) and oxidative stress analyses.

General commentaries

- Line 24–26: excessive use of parenthesis () in one single phrase.
- Line 26–28: Here the main result of the manuscript: "heat wave produces brain oxidative damage and in absence of water these heat waves can damage the liver and the kidney", but <u>the authors do not show the information of body temperature or dehydration</u>.
- Line 46–47. which measurements did the authors use to determine the temperatures in the local habitat of the specimen?
- line 58–59. Some Keywords are repeated in the title
- Until line 72, I did not read the connection between sublethal effects of heat waves related to oxidative damage rise. Literature review about this topic is extremely necessary.
- Lines 95–101. Although it is true that oxidative stress produces damages in several subcellular structures and disruption in some metabolic

pathways, it is also true that the production of low quantities of ROS is very important triggers for strategies against oxidative damages. I strongly recommend that the authors read papers such as: DOI: 10.1016/j.freeradbiomed.2015.07.156 or DOI: 10.1016/j.cbpa.2019.04.004, and especially have a reflection about the concept of "hormeses" and its importance for the knowledge of the biochemistry ecology (related to oxidative balance) of wild animals of extreme environments (DOI: 10.3389/fphys.2018.00945). Since ~2000, to consider the ROS production as strictly "a problem" could be an idea out of date.

- Lines 104–106. Several studies evaluate measurements of oxidative stress in a variety of tissues as a consequence of exposure to different thermal regimes in numerous animals. I did not understand if the authors referred to these studies in *Rhabdomys dilectus* ?.
- Lines 113–123. I find unnecessary and too long justification of why the authors choose the brain, kidney, and liver as tissue models for the redox tests.
- Lines 174–179. The authors deserve a high praise for the methodology chosen for the temperature measurement. Even more by the hydration control done. However, I did not find these records in the results or discussion.
- Lines 206–208. How authors can be sure that do not exist "heat stress" in animal field conditions under the influence of a daily temperature cycle from an average summer day? I did not find any data about the heat stress of R. dilectus in the field. In my opinion, the author still can use the "Control" group as they delimitated, but they cannot assume the absence of heat stress in the field for the model species without data related.
- Line 221. Climate condition? Climate condition means all the conditions associated with environmental factors in the habitat of the species. In this experiment, the authors just controlled the temperature (it does not mean that they had little work in the design of the experiment). I felt some confusion in this section with the terms CLIMATE VS WEATHER.
- Line 235. From my point of view, **here is the biggest problem in the experimental design of these experiments**. It is well known that the use of volatile anesthetics, as "isoflurane", induce oxidative stress (https://www.ncbi.nlm.nih.gov/pmc/articles/PMC4458520/), that is why I will ask the authors an explication of how can they be sure that the result obtained (by temperature treatments) are not influenced by the overdoses of isoflurane.
- Line 419. Table 1 is unnecessary. If the authors want to use a table, I believe that it is a better idea to show a summary of all the oxidative stress and oxidative damage parameters in one single table and summarizing paragraphs of the results.

• Lines 434–440. I did not find where the authors showed the result that underpins this part of the discussion (the thermal biology and water balance discussion). Mainly, I am very curious to know the data related to "heat gain

and high heat loads in tissues”. Without these supports, all the discussion loses its validity.

- Lines 467–470. Until these lines, I had not seen any result about water balance in this manuscript. First of all, the percentage of body mass lost have to be in the results section, not here in the discussion. A complete water balance analysis (statistical analysis) is required for this manuscript to substance this discussion. Finally, I require the authors a better explanation about how a slight body mass lost (~2% of body mass lost) can be related to a very grave physiological state as is the hyperosmolarity (talking in terms of magnitude).
- Lines 490… Climate change, related to temperature? Climate changes it much more than global warming. I assume that the authors are talking about some “warming effect”. I suggest moderation in this paragraph, especially because the authors do not show results about behavioral or physiological thermoregulation (or thermoconformation) of the species in its microhabitats. I am not discarding the possibility, but the authors do not show data on the environmental physiology of the species to support this kind of discussion.
- Line 500: “animals from arid regions, which are already living at their physiological limit”. I strongly suggest to the authors think about this phrase. I felt it as an “anthropocentric view” totally outside of actual knowledge of the environmental physiology of animals of extreme environments. I highlight this phrase because the authors do not show data that support that their model species (or other animals from arid regions) is at the edge of their physiology. I certainly do not rule out this possibility with some species, but in the light of the actual literature of environmental physiology, it is a mistake to generalize it.
