# Supplementary material for: Heat and dehydration induced oxidative damage and antioxidant defenses following incubator heat stress and a simulated heat wave in wild caught four-striped field mice Rhabdomys dilectus
Source: PLoS One. 2020 Nov 13;15(11):e0242279. doi: 10.1371/journal.pone.0242279 (PMC7665817; doi:10.1371/journal.pone.0242279)
Supplement: Attachment — Submitted filename: Response to Reviewers.docx [file pone.0242279.s005.docx]

We wish to thank all the reviewers for their comments and their appraisal in addressing their initial concerns and improve the manuscript.

Response to Reviewer 1:

Reviewer #1: The authors accepted all the recommendations and resolved the doubts I raised in my first review. I believe that the article gained much more scientific rigor and is a good contribution to understanding the adaptive redox processes of animals that survive in extreme conditions. Nevertheless, I should like to comment one detail that do not fully satisfy me. The point is the change values of the body mass of the treatment vs the control group. Although there is a significant difference (F1 = 23.47, p = 0.001), the values present a lot of intrinsic variation in itself (differences between the control and treatments of 0.45 ± 0.66%; see S2 TABLE). Having negative treatment vs control values means that some mice were hydrated instead of dehydrated, which is not addressed by the authors (See new table S2) Outside of this points, I consider that the manuscript improved a lot from its first version.

Lines 423-426: As the authors currently understand the control group had a positive body mass change 0.45 ± 0.66%, whereas the treatment group had a negative change. This suggests a gain in body mass (for the control group) suggesting individuals were hydrated. This can be explained as body mass measurements were taken before the saline injection. The saline injection likely increased the body mass to some extent, which was observed post-experiment. Due to the significant water losses in the treatment group, all individuals demonstrated decreased body mass changes, whereas the control group demonstrated some individuals who had a net gain in body mass.
